# Anaemia

**Published:** 1898-02

**Authors:** 


### ANAEMIA.

### WHENCE COME THE RED CORPUSCLES?

One author says—speaking broadly—that in the lymphatic network the germ springs which grows into a hæmatoblast; others say the germ of the hæmatoblast takes origin in, and is vitalized by, special functions belonging to bone-marrow; another claims that certain great glands, namely, the liver and spleen, are probably the legitimate parents of the little brilliants we recognize as hæmatoblasts; i. e., embryonic discs. After much perplexed study upon cellular pathology, evolution, and so forth, we believe the several anatomical structures possess within themselves provisional reproductive powers, and add, one and all, a quota of fecundity to the great column of blood, receiving in turn warmth, nutrition, and the principles which foster perpetuation. It is, therefore, seen that the entire body is, in great measure, involved in the conception and evolution of hæmatoblasts. We do not attempt to deny that there are special hæmatopoietic centers; in truth, we are inclined to believe that in the lymphatic network exists probably the most favorable and nurturing bed for the generation and protection of the hæmatoblast. These little brilliants evolve into red corpuscles —the length of time required is not known—they become, however, in the course of time, and under favorable nurturing circumstances, full blown discs, provided with a special membrane containing globuline, cholesterine, leceithine, albuminoid matter, and a very complex red matter, which is azotized and crystalizaable, composed chiefly of iron—the oxy-hæmoglobin. Upon the richness of the blood in the latter product hangs its life-giving functions.

### WHENCE COMES DISEASE OF RED DISCS?

Anæmia of every form—and there are many—has its origin in some primary affection; i. e., disease of the hæmatoblasts, or of the hæmatopoietic centers—which leads on to poverty of the blood. Science attains its highest object when it correctly directs the healthful growth and propagation of the solid constituents of the blood. It requires an abundance of pure air every day; exercise of all the muscles and faculties of the general economy is demanded; sunlight is of great value; and a free supply of soluble, nutritious aliment is indispensable. There are those who receive day by day none of the requisite factors in full measure; and they become in the course of time hydræmic, polyæmic, or chlorotic. The post-hemorragic variety—known as true anæmia—is due, of course, to sudden loss of blood from severed vessels.

### THE MODE OF DETECTING ANÆMIA.

Many patients are hopelessly afflicted who believe themselves in perfect health. Many are past relief when their ulterior physical appearance would not indicate the same even to the eye of a physician. To ascertain reliable information, put a fresh drawn drop of blood under microscope and observe the form and estimate the number and quality of red discs as compared to a drop of normal blood; note particularly the number of hæmatoblasts in the field relative to grown globules; make a qualitative estimation of hæmoglobin as compared to the number of red corpuscles indicated. If the little brilliant hæmatoblasts are scanty, and the hæmoglobin deficient, the case is serious; if the brilliants are absent the case is hopeless, and a fatal issue may with safety be prognosed; since if the hæmatopoietic centers are diseased to the extent that no hæmatoblasts appear in the field the blood must of necessity quickly become fatally impoverished, and the patient must eventually die of pernicious anæmia; hence the necessity of careful examination in due time.

### THE TREATMENT OF ANÆMIA.

There are two lines to pursue, namely, (1) the administration of scientific pharmaceutical remedies, and (2) the enforcement of systematic dietary and exercise. Some of the most distinguished authors give prominence to, and discuss first, the pharmaceutical side of the question. They claim that the most profitable step in the course of treatment is to settle upon an acceptible mode of supply to the pale economy its deficient quantities of iron in the red discs and in the oxy-hæmoglobin—iron being the most important part of these respective substances, and the oxy-hæmoglobin being the veritable factor in the respiratory power of the blood. No one understands the process by which iron enriches blood and imparts vigor to the body, i. e., its pharmaco-dynamic action is not yet satisfactorily explained; but every clinician has his clinical knowledge, and this bears more reliable testimony than theories. Iron was first prescribed because, as a metal, it represented force; its general use has been continued because of its discovery in normal blood cells, and in many of the secretions; for instance, one observer found three centigrams in one day's secretion of gastric juice, and one centigram more in bile of the same period; he found it also in tears and saliva. The mode of entrance of iron into the blood, and its physiological action, has induced great controversies, and is now considered a classical subject. One would say—he who favored the liver as a special hæmato-poietic center—that the intestines partially absorbed iron, but that it was not until it reached the hepatic cells, and met with biliary influences, that it became chemically mixed with the solid elements of the blood; another would say the favoring conditions which amalgamated iron and blood were harbored in the lymphatic system; still another would claim that the stomach juices, the bile, and the secretions of the ilium, are all concerned in the process of ingress. The writings of every one will show that heretofore it has been difficult to get the full effect of the drug short of the superinduction of very disagreeable symptoms somewhere along the alimentary tract; therefore it is easily seen that the most important point in reference to its administration is the avoidance of gastric and intestinal irritability; for if such conditions develop, the virtue of the remedy is more than counterbalanced by dyspeptic and other deleterious sequella, which immediately follow. When this order of circumstances is observed to prevail, the system, instead of deriving strength from our endeavors, becomes surely more weakened every day, and will never regain vigor until the gastric and enteric mucosa become healed with proper rest and remedies. The selection, then, of a non-irritating, absorbable preparation is important, and may be the means of forestalling dyspepsia, and the like. We note that Dr. Love, of St. Louis, reports fifty cases of globular anæmia cured by the use of pepto-mangan (Gude), and without the supervention of stomach derangement. This is a record of great strength. Dr. Summa observed *seven per cent.* volume increase of red corpuscles *in eight days.* This is remarkable. There were eight cases treated at Bellevue, in which there was an average increase of more than two per cent. of hæmoglobin, and approximately an increase of red discs to the enormous extent of one million two hundred and fifty-eight thousand. We suppose, though it is not so stated, that this record refers to one cubic millimetre; and we imagine the patients were at least one month under treatment, though the date is likewise unstated. These observations are of the very greatest value, and will fall like balm into the hearts of physicians who have repeatedly become vexed over the aggravating and ofttimes futile use of muriate tincture, exsiccated sulphate, and reduced iron by hydrogen.

